# Supplementary material for: A multi-omics approach to maize (Zea mays) tassel development
Source: BMC Plant Biol. 2026 Jul 8;26:1180. doi: 10.1186/s12870-026-09413-w (PMC13355352; doi:10.1186/s12870-026-09413-w)
Supplement: Supplementary file 1 — Supplementary Material 1: Supplementary figure 1. Sequences of probes for RNA in situ hybridization assays. Supplementary figure 2. Principal component analysis of normalized RNAseq counts. Supplementary figure 3. Heatmap of expressed genes. Supplementary figure 4. Heatmap of K-means clustering. Supplementary figure 5. K-means clustering of all expressed genes per cluster. Supplementary figure 6. Expression patterns of selected genes. Supplementary figure 7. Expression patterns of meiosis-associated genes. Supplementary figure 8. Expression patterns of selected differentially expressed genes. Supplementary figure 9. Principal component analysis of miRNAseq counts. Supplementary Figure 10. K-means clustering analysis of highly variable proteins. Supplementary Figure 11. Go term analysis of proteins. Supplementary figure 12. Expression pattern of GSL4. [file 12870_2026_9413_MOESM1_ESM.pdf]

Supplementary Figures 1-12

Article Title: A multi-omics approach to maize (*Zea mays*) tassel development

Finn Hartmann[1], Sandra Mathioni[2], Atul Kakrana[3], Blake C. Meyers[2,4,5], Virginia Walbot[6], Karina van der Linde[1*]

[1]Plant Cell Biology, Biochemistry, and Biotechnology, University of Regensburg, Regensburg, Germany

[2]Donald Danforth Plant Science Center, St. Louis, Missouri, USA

[3]Data Science Institute, University of Delaware, Newark, Delaware, USA

[4]Center for Bioinformatics and Computational Biology, University of Delaware, Newark, Delaware, USA

[5]The Genome Center, University of California, Davis, Davis, California, USA

[6]Department of Biology, Stanford University, Stanford, California, USA

***Correspondence:**

Karina van der Linde: karina.van-der-linde@ur.de

a

```
  1 taccgagggt cgttcgaacg ccaccagcag cggaaccatc gcagcgagga ggacgagtgg tggtcgttgt
 71 ggttcgaacc ggacaagcac gagccggtcc gacgaggccc gcggatgggt ggtgcccgag gaggcggcgt
141 ggtctagcag ctggagcggt ttctgacgcc cccgcgcacg ctgcacgcca cgccgcgcgt gagcgcgttc
211 ttgtagacgt gggcccgcac ggagttcacg acgccgcaga cggcgacgca cggccgcccg tgacggccgt
281 tggtcgtctg cacgccgttc acgatgtggc tgacctggtg cgtgccgttg ttctggttca cgggcact
```

b

```
  1 actgagtcgg aacgagaaag agttgccaaa atggtgggag ttgtttctgt attcccaaac aagaagttac
 71 aacttcaaac gacgacgtct tgggacttca tggggttaaa ggaaggaata aagacgaaga ggaaccctac
141 cgtcgaaagt gatacaatta ttggagttat cgacagtggg atcacgccgg aatctcagag cttttcggac
211 aaaggctttg gtcctcctcc tcagaaatgg aaaggtgttt gttccggcgg caaaaacttc acatgcaaca
281 acaagttgat tggggcaaga gactacacaa gcgaaggtac tagggacatg gacggacacg gtacacacac
351 tgcgtccacg gcggctggaa acgcagttgt ggacgcaagc ttctttggaa tcggcaatgg aaccgtaaga
421 ggtggtgttc cagcctctag agtagccgct tacaaagtct gcaccccgac agggtgtagt tcggaagctc
491 tactgtctgc attcgatgac g
```

Supplementary Fig 1. Sequences of probes for RNA *in situ* hybridization assays. a Anti-sense probe sequence used to detect *GSL4* expression. **b** Sequence of the random probe used in RNA *in situ* hybridization assays as control.

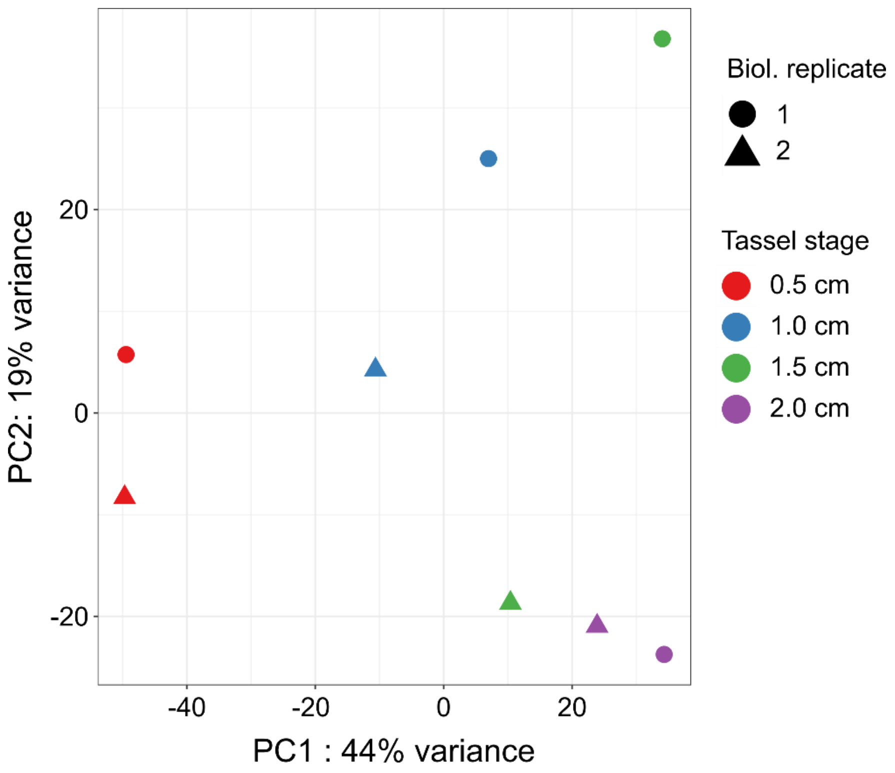

Supplementary Fig 2 Principal component analysis of normalized RNAseq counts. Normalized RNAseq counts of two independent biological replicates (biol. replicate 1 = circle, biol. replicate 2 = triangle) were analyzed per tassel stage. Tassel stages are color coded (0.5 cm = red,1.0 cm = blue, 1.5 cm = green, 2.0 cm = purple).

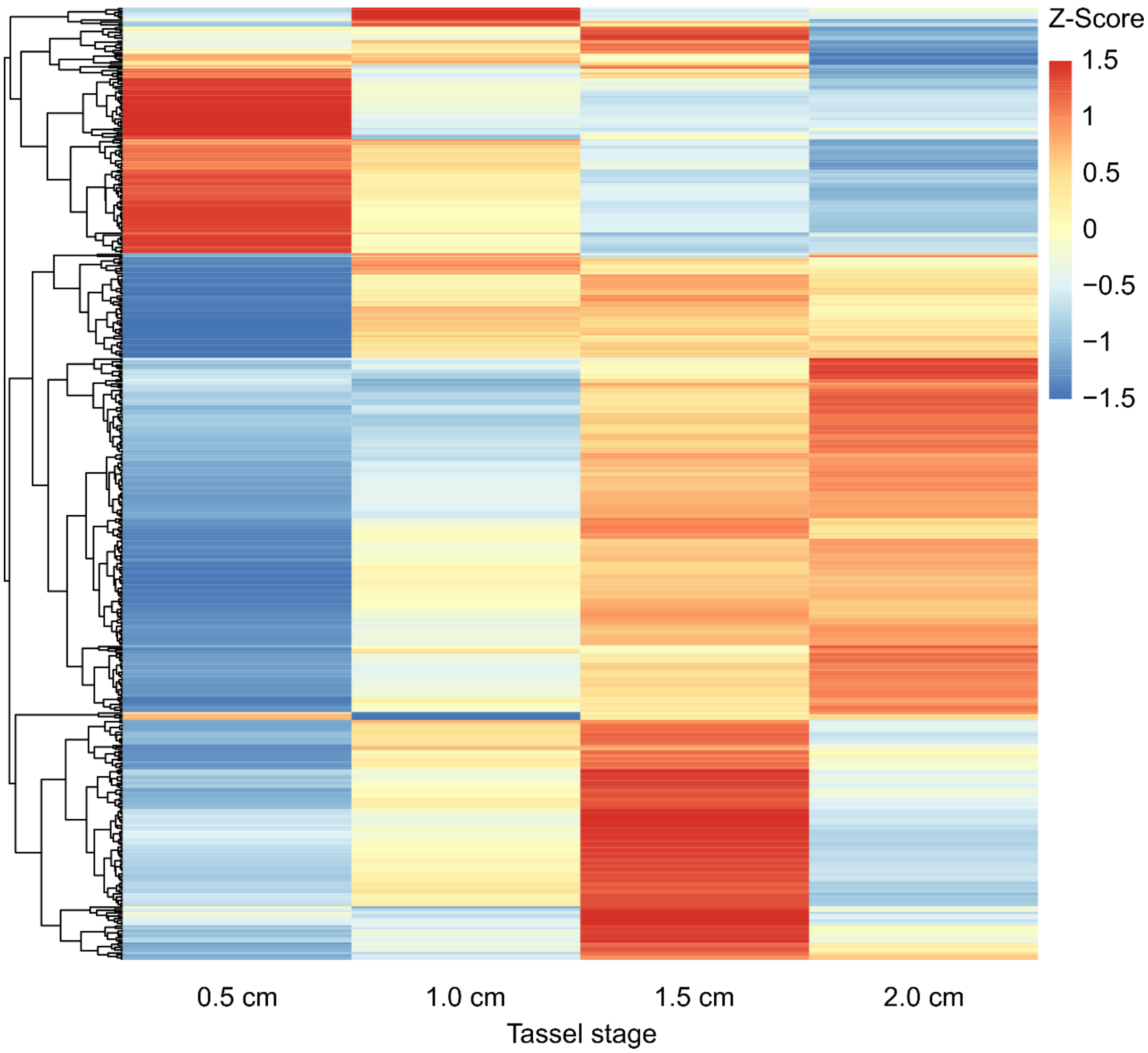

Supplementary Figure 3 Heatmap of expressed genes. Z-scores per tassel stage were calculated and hierarchically clustered for each normalized gene expression count.

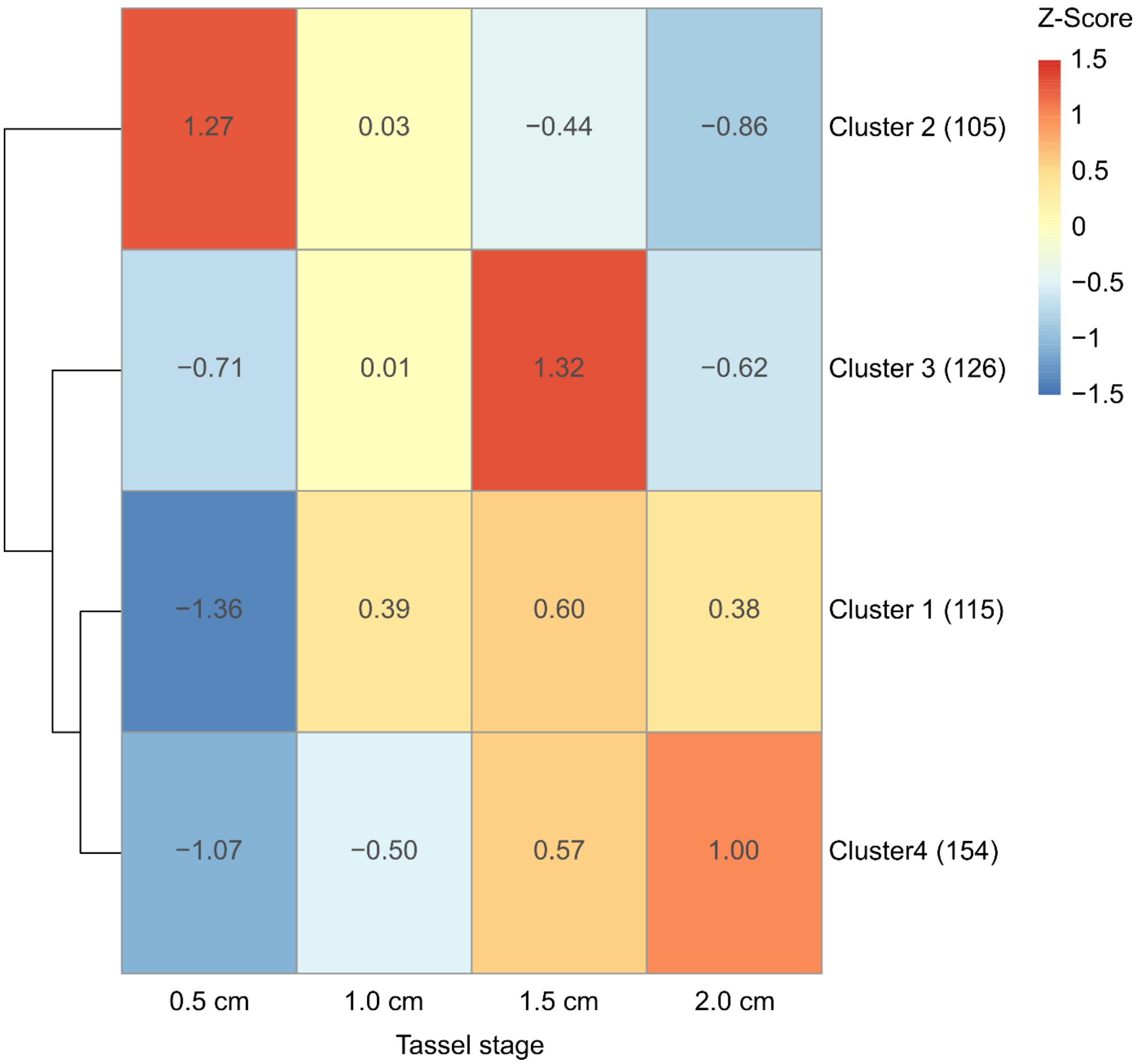

Supplementary Figure 4 Heatmap of K-means clustering. The best number of K-means clusters was calculated, and the corresponding Z-scores were clustered hierarchically and by tassel stage

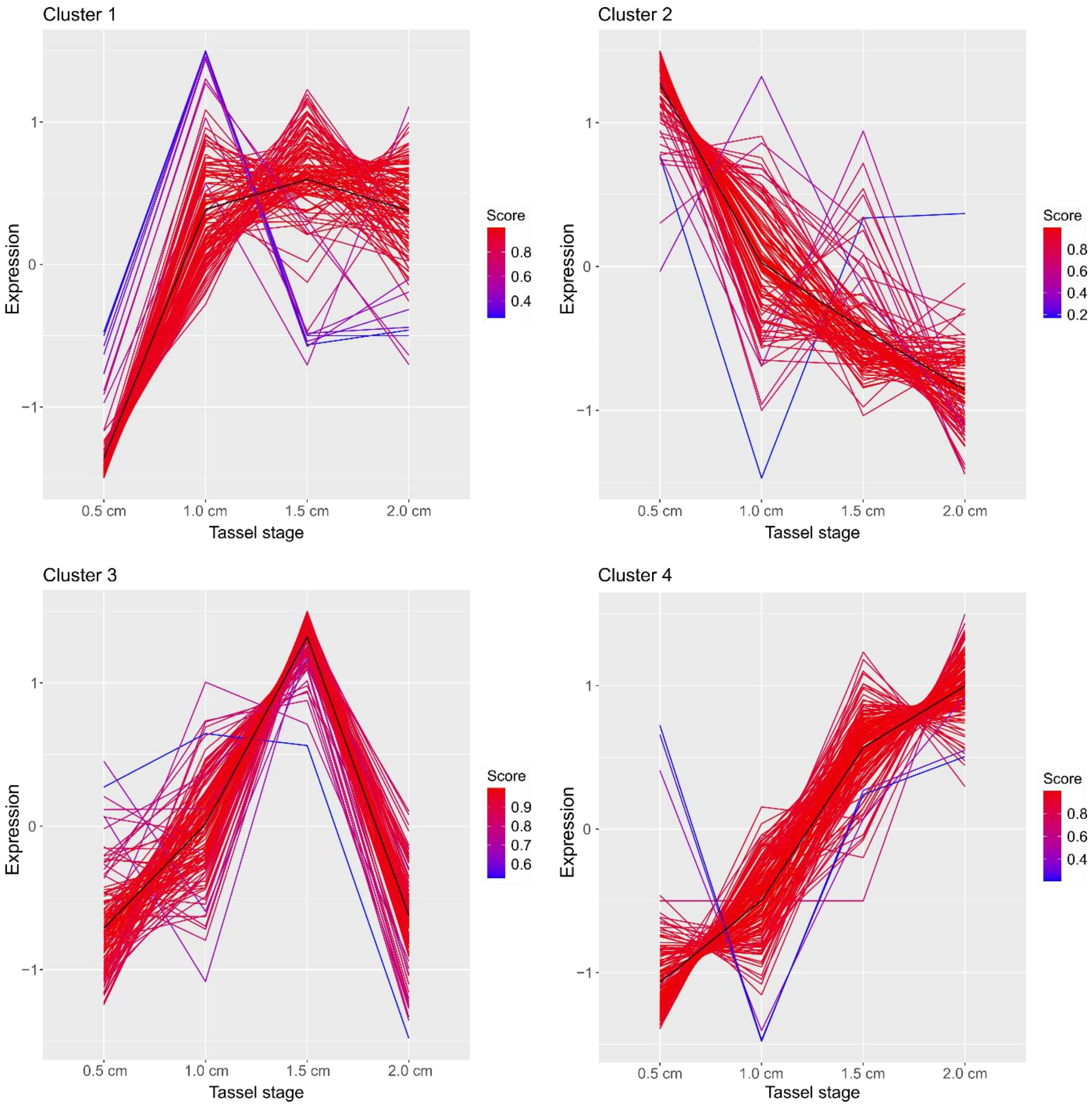

Supplementary Figure 5 K-means clustering of expressed genes per cluster. Centroids (mean value of normalized expression values per cluster, black) and all expressed genes per cluster are plotted over tassel stages**.** For each expressed gene the fit score is indicated by color.

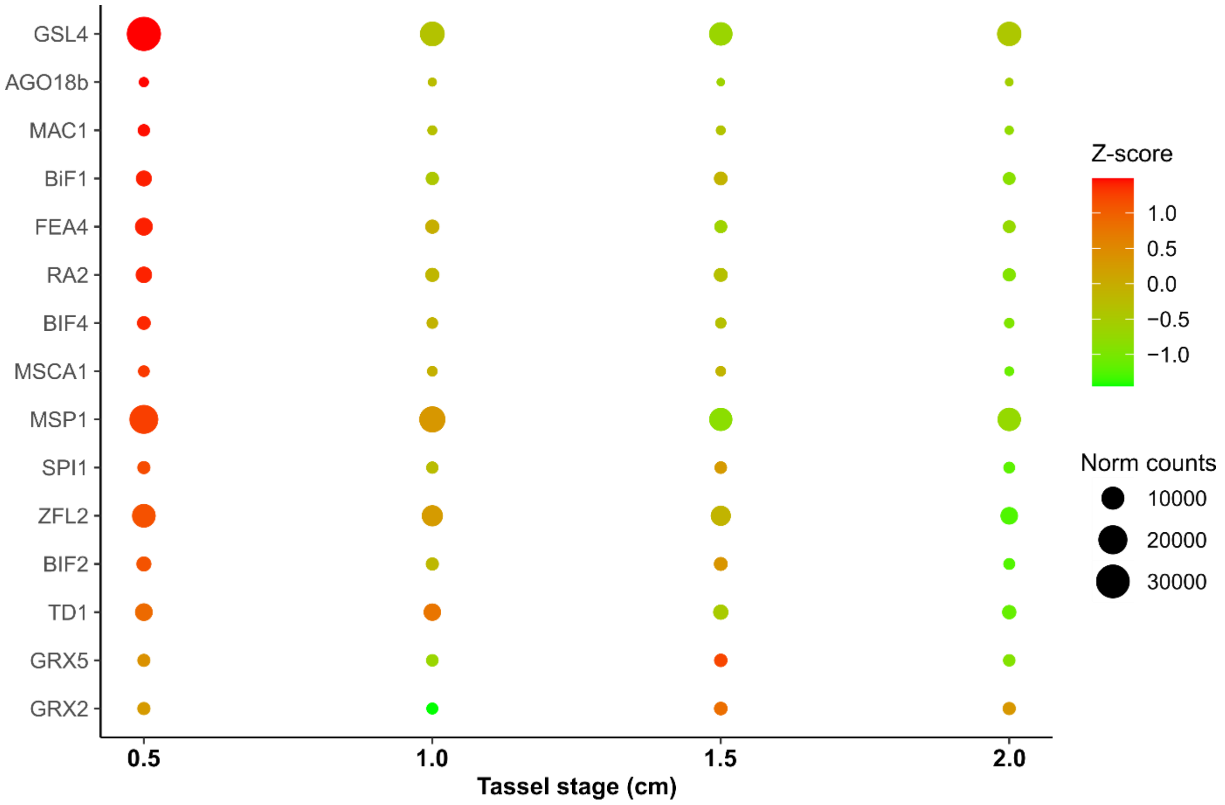

Supplementary Figure 6 Expression patterns of selected genes. Mean DESeq2-normalized counts (library-size–normalized expression, Norm counts) and Z-scores for *AGO18b*, *BIF1,2,44*, *ZFL2*, *FEA2*, *FEA4, MSCA1*, *GRX2*, *GRX5*, *MAC1, MSP1, GSL4, RA2 and SPL1* along the four sampled tassel developmental stages (0.5 cm, 1.0 cm, 1.5 cm, 2.0 cm).

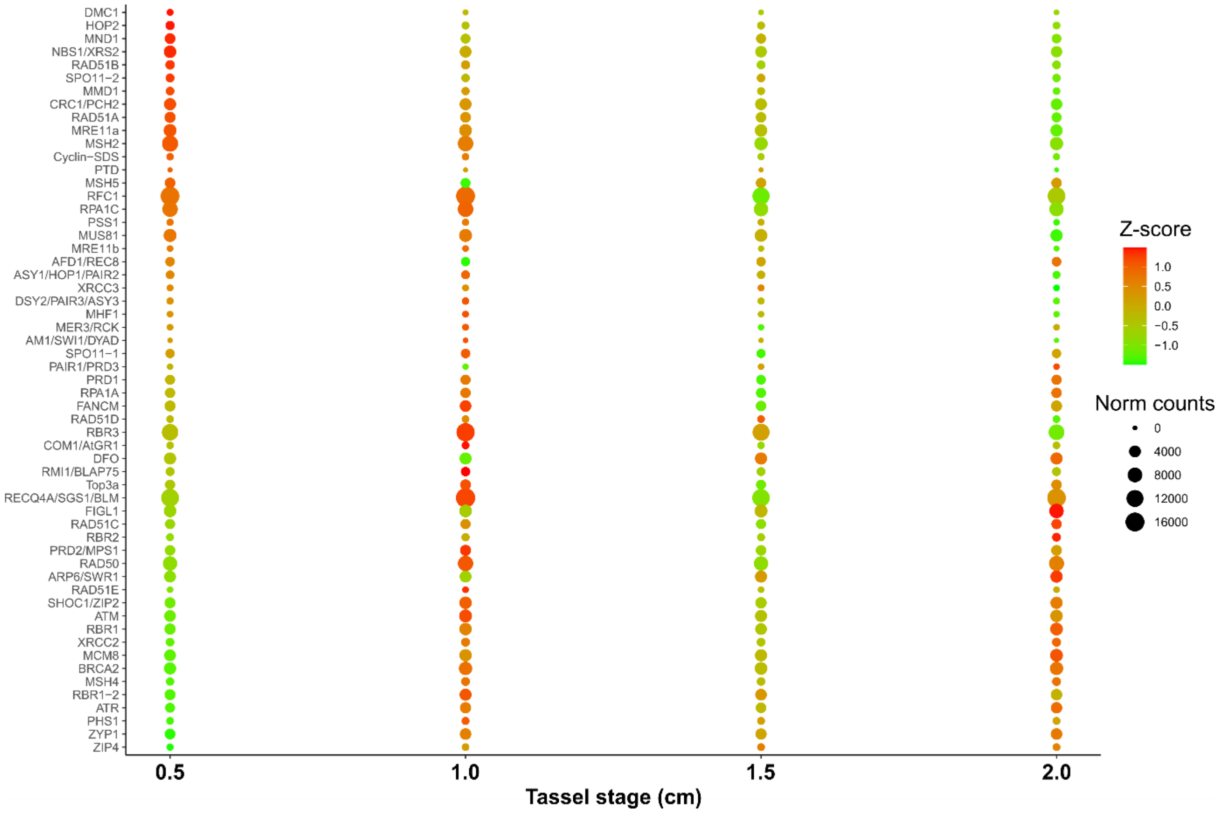

Supplementary Figure 7 Expression patterns of meiosis-associated genes. Mean DESeq2-normalized counts (library-size–normalized expression, Norm counts) and Z-scores for previously identified meiosis-associated genes [52].

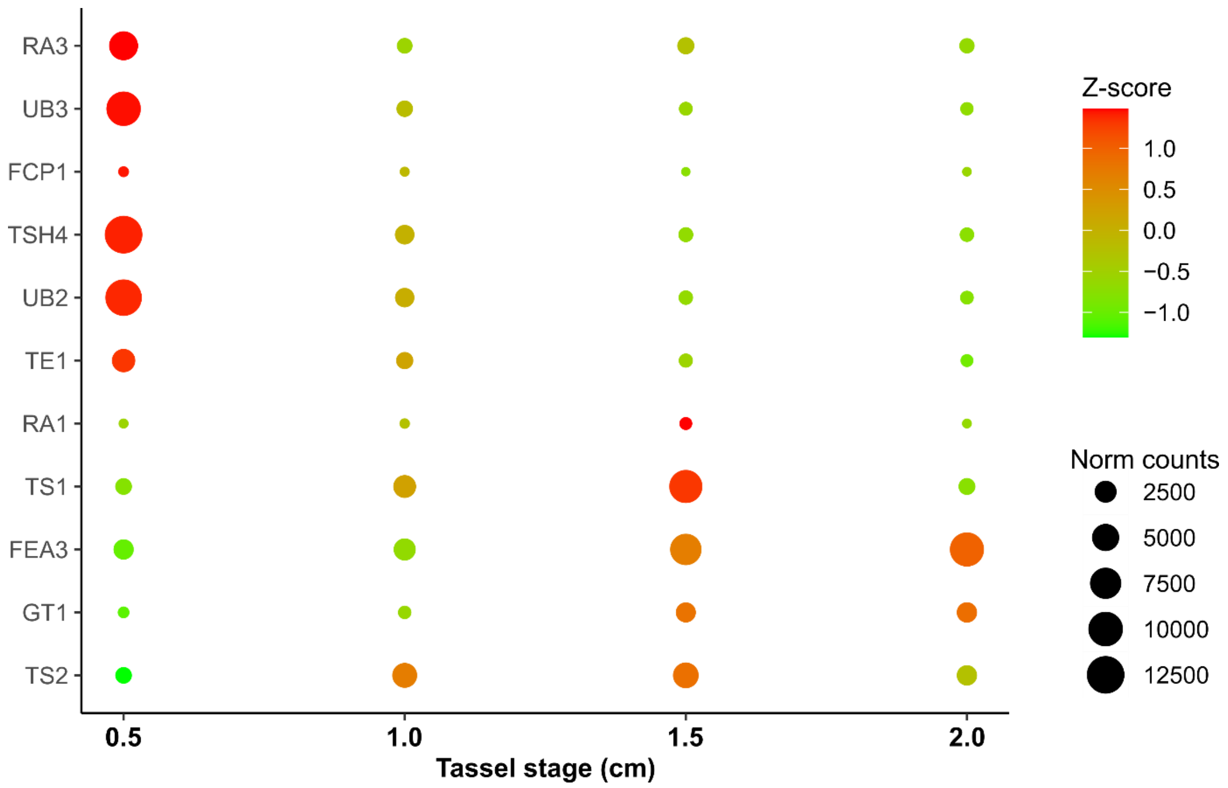

Supplementary Figure 8 Expression patterns of selected differentially expressed genes. Mean DESeq2-normalized counts (library-size–normalized expression, Norm counts) and Z-scores for *FCP1, RA1, TS1; TS2, GT1, FEA3, RA3, TE1, UB2, UB3, TSH4*.

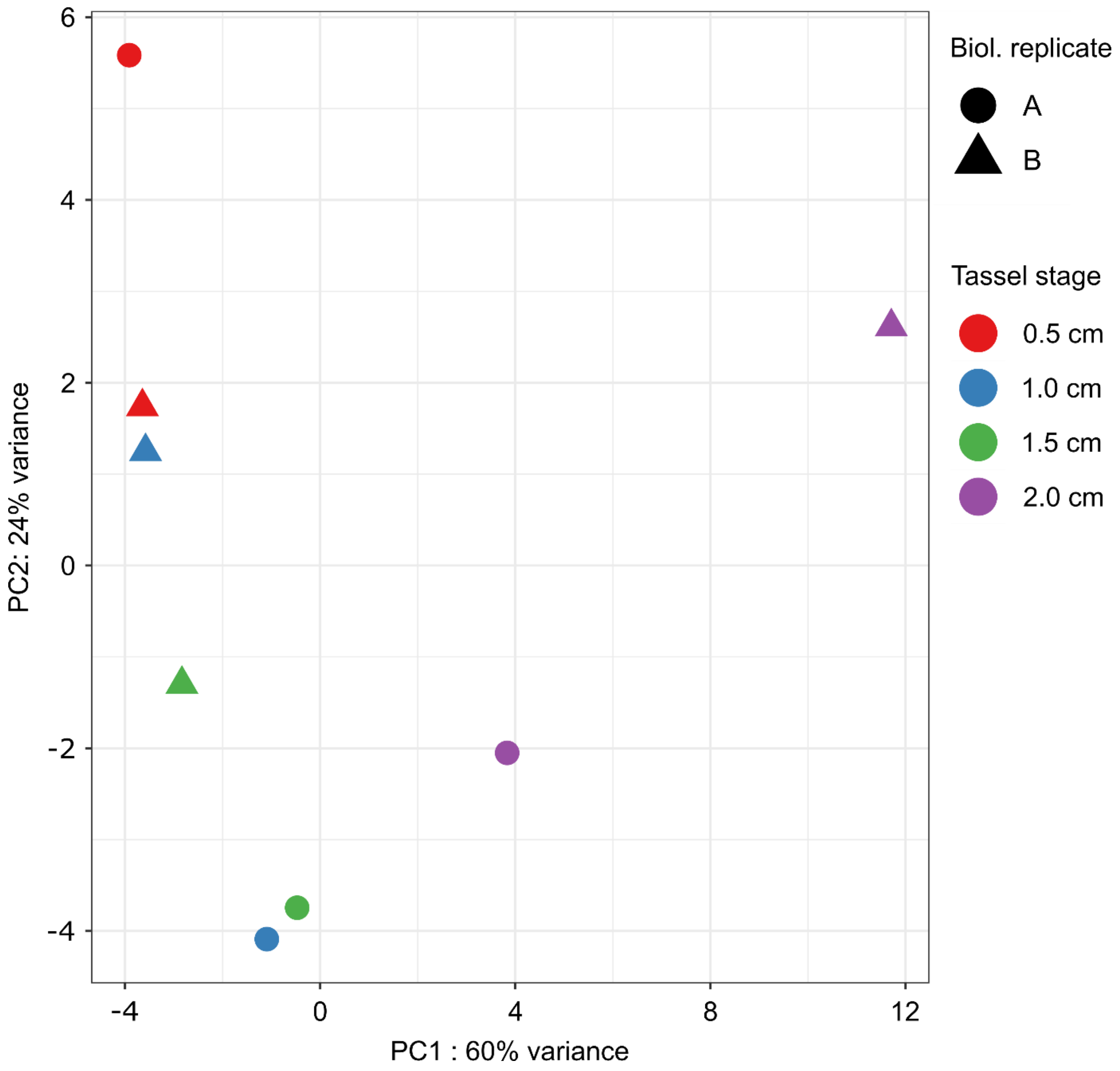

Supplementary Figure 9 Principal component analysis of miRNAseq counts. Normalized miRNA counts of two independent biological replicates (biol. replicate 1 = circle, biol. replicate 2 = triangle) were analyzed per tassel stage. Tassel stages are color coded (0.5 cm = red, 1.0 cm = blue, 1.5 cm = green, 2.0 cm = purple).

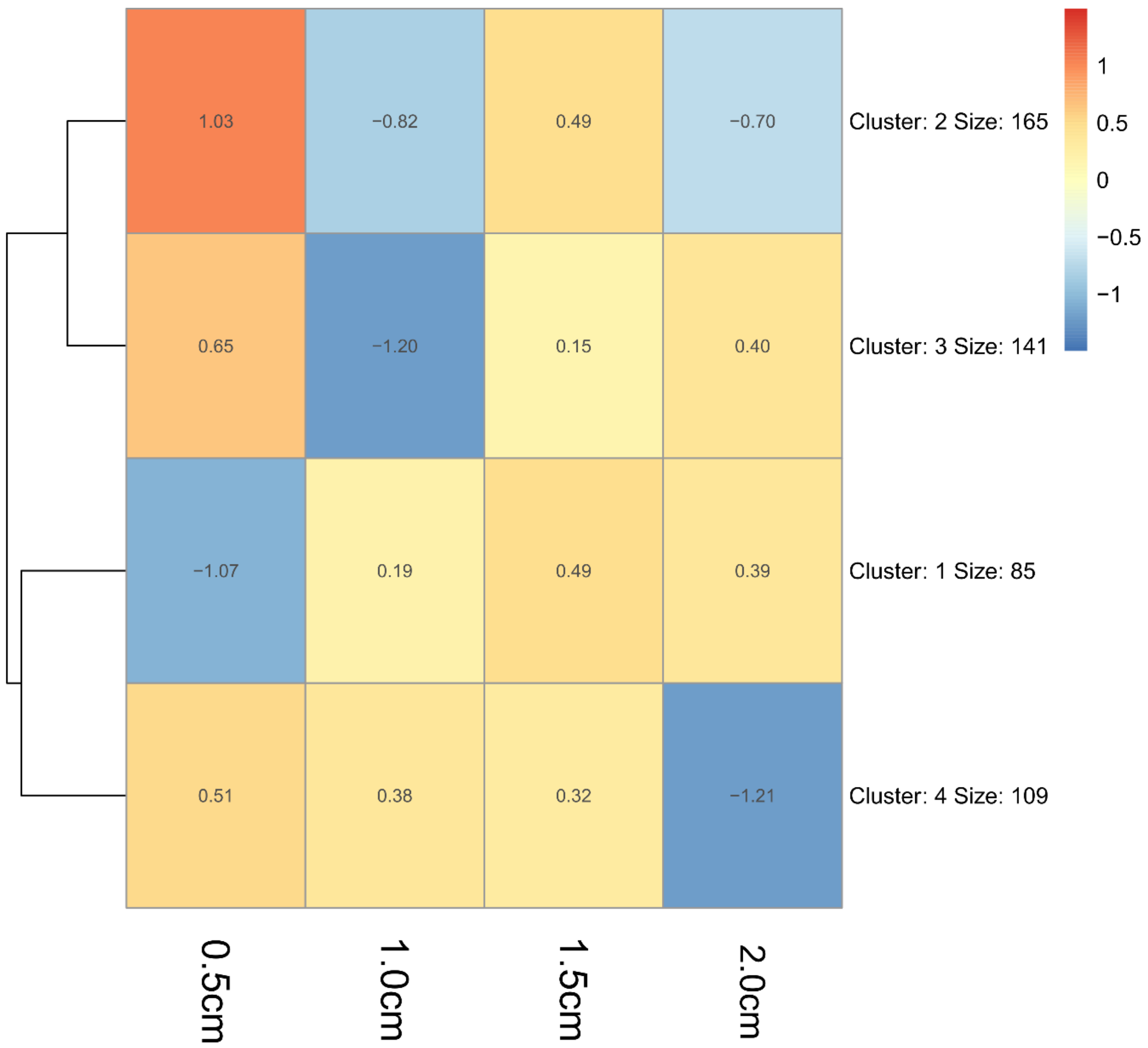

Supplementary Figure 10 K-means clustering analysis of highly variable proteins. The heat map was generated using the top 500 proteins exhibiting the highest row variance across the experimental timeline with coloring indicating Z-scores (row-wise normalization).

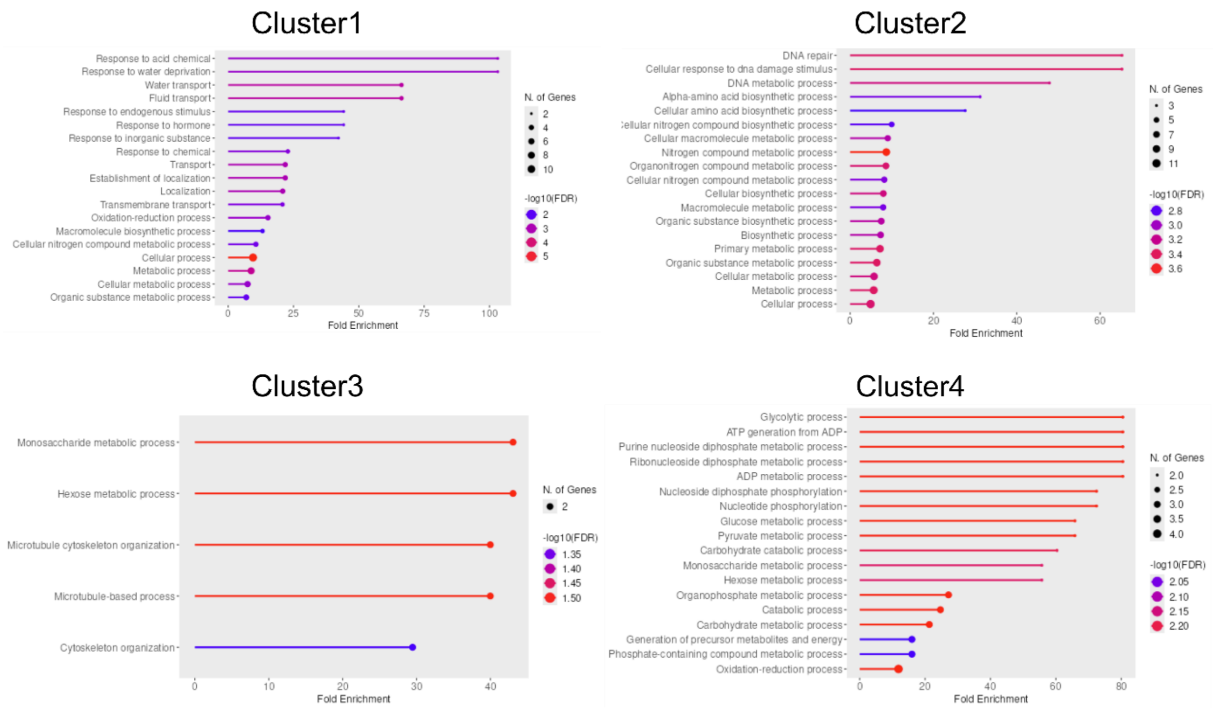

Supplementary Figure 11 Go term analysis of proteins. Go term analysis of proteins for each cluster in Supplementary Figure 10. GO terms were selected by FalseDiscoveryRate (FDR), sorted by fold enrichment and number of genes are displayed as respective circle radius.

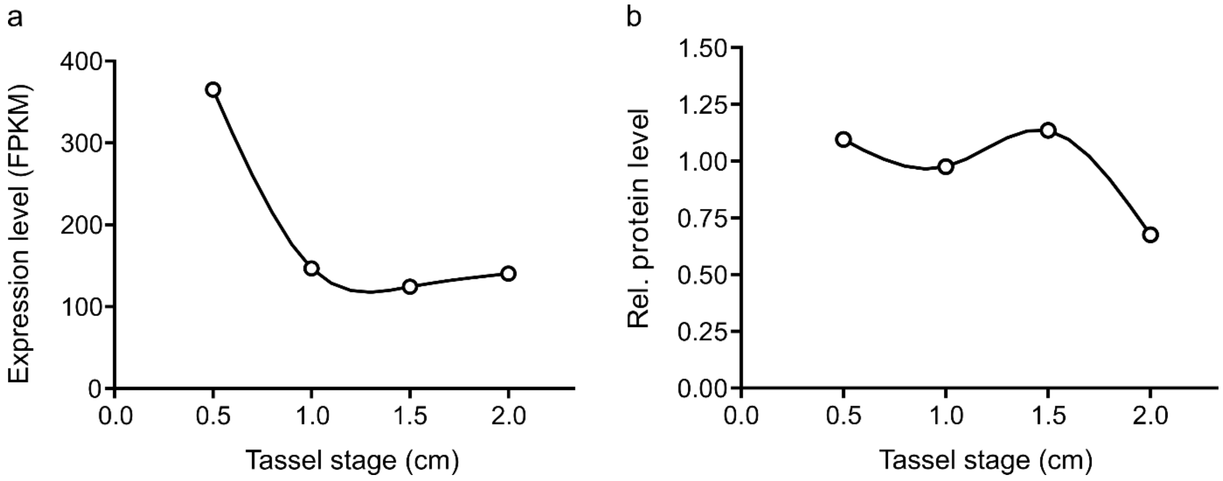

Supplementary Figure 12 Expression pattern of GSL4. a The expression pattern of GSL4 is given based on the transcriptomic data (in FPKM) and **b** based on the proteomics data (in relative protein level) for the four different tassel stages.
